# Supplementary material for: Modulation of Neurexins Alternative Splicing by Cannabinoid Receptors 1 (CB1) Signaling
Source: Cells. 2025 Jun 25;14(13):972. doi: 10.3390/cells14130972 (PMC12249465; doi:10.3390/cells14130972)
Supplement: Supplementary file 1 [file cells-14-00972-s001.zip › cells-3658259-supplementary.pdf]

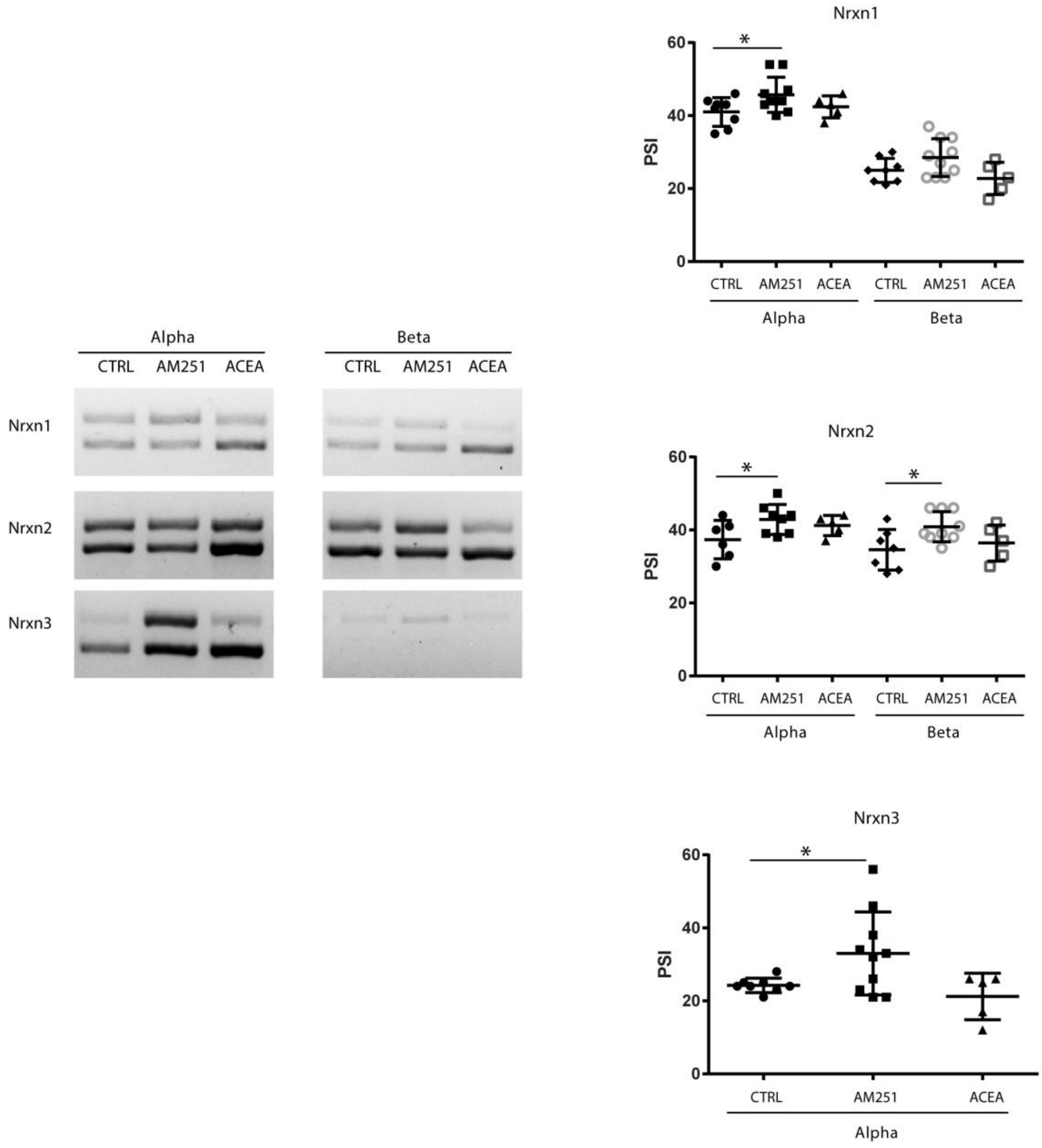

Figure S1. Splicing pattern of α/β Nrxns after CB1 receptors stimulation.

A) RT-PCR analysis performed with selective primers to discriminate α and β isoforms of Nrxns (1-3) in hippocampal slices after stimulation for 6h with ACEA or AM251. B) Relative scatter plots show the PSI, calculated by densitometric analysis (Image Lab 6.0) of Nrxns (1-3) splicing pattern in hippocampal slices. AM251 treatment induced higher level of +SS4 (inclusion) in α and β isoforms. Hippocampal slices treated

only with vehicle were used as control. Results were obtained from (5-10) samples selected from n=2 independent experiments. *p < 0.05; Error bars represent SD.

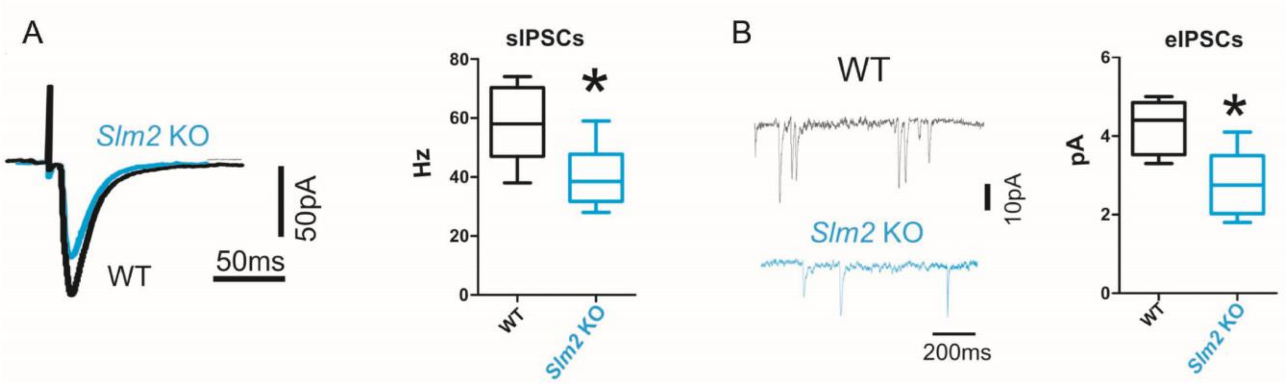

Figure S2. ***Slm2* ko electrophysiological characterization of GABAergic input.**
Representative traces of spontaneous (A) and evoked IPSCs (B) recorded from WT (black) and *Slm2* ko mice(blue). Box plots show that *Slm2* ko mice have a significant reduction of both evoked IPSCs amplitude and spontaneous IPSCs frequency.
